# HCV Infection Enhances Th17 Commitment, Which Could Affect the Pathogenesis of Autoimmune Diseases

**DOI:** 10.1371/journal.pone.0098521

**Published:** 2014-06-06

**Authors:** Yasuteru Kondo, Masashi Ninomiya, Osamu Kimura, Keigo Machida, Ryo Funayama, Takeshi Nagashima, Koju Kobayashi, Eiji Kakazu, Takanobu Kato, Keiko Nakayama, Michael M. C. Lai, Tooru Shimosegawa

**Affiliations:** 1 Division of Gastroenterology, Tohoku University Graduate School of Medicine, Sendai City, Miyagi, Japan; 2 Department of Microbiology and Immunology, Keck School of Medicine, University of Southern California, Los Angeles, California, United States of America; 3 Division of Cell Proliferation, Tohoku University Graduate School of Medicine, Sendai City, Miyagi, Japan; 4 Department of Virology II, National Institute of Infectious Diseases, Shinjuku, Tokyo, Japan; 5 China Medical University, Taichung, Taiwan; University of Washington, United States of America

## Abstract

**Background:**

Various kinds of autoimmune diseases have been reported to have a significant relationship with persistent hepatitis c virus (HCV) infection and Th17 cells. Previously, our group reported that the existence of HCV in T lymphocytes could affect the development of CD4^+^ helper T cells and their proliferation, in addition to the induction of immunoglobulin hyper-mutation.

**Methods:**

Therefore, we analyzed the relationship between persistent infection of HCV and the mechanism of Th17 cell induction *ex vivo* and *in vitro*.

**Results:**

The prevalence of autoimmune-related diseases in chronic hepatitis c patients (CH-C) was significantly higher than in other types of chronic hepatitis (hepatitis B and NASH). A significantly higher frequency of IL6 and TGF-β double-high patients was detected in CH-C than in other liver diseases. Moreover, these double-high patients had significantly higher positivity of anti-nuclear antibody, cryoglobulinemia, and lymphotropic HCV and higher amounts of IL1-β, IL21, IL23. In addition to the previously reported lymphotropic SB-HCV strain, we found a novel, genotype 1b lymphotropic HCV (Ly-HCV), by deep sequencing analysis. Lymphotropic-HCV replication could be detected in the lymphoid cells with various kinds of cytokine-conditions including IL1β, IL23, IL6 and TGF-β in vitro. Infection by HCV could significantly enhance the development of Th17 cells. The HCV protein responsible for inducing the Th17 cells was HCV-Core protein, which could enhance the STAT-3 signaling and up-regulate the expression of RORγt as a Th17 master gene.

**Conclusion:**

Infection by lymphotropic HCV might enhance the Th17 development and contribute to understanding the pathogenesis of autoimmune-related diseases.

## Introduction

Cellular and humoral immune responses to HCV play an important role in the pathogenesis of chronic hepatitis, HCC and B-lymphocyte proliferative disorders including mixed cryoglobulinemia, a disorder characterized by the oligoclonal proliferation of B cells [1]–[5]. B cell activation and/or dis-regulation could originate as a result of HCV binding to CD81 tetraspanin molecule or as a consequence of its ability to replicate in B lymphocytes[6]. It has been reported that HCV could infect B lymphocytes[7]–[9]. We previously reported that HCV-replication in B lymphocytes could induce immunoglobulin hypermutation and reduce the affinity and neutralizing activities of antibodies against HCV envelope protein[5]. On the other hand, the hypermutation of immunoglobulin might induce autoantibodies that contribute to the immunopathogenesis of autoimmune diseases, since various kinds of autoimmune diseases were reported to have a significant relationship with persistent HCV infection [10]–[12].

Previously, our group reported that the existence of HCV in T lymphocytes could affect the development and proliferation of type 1 T helper (Th1) cells[3], [4], [13]. Other groups have also reported the existence of HCV in T lymphocytes[14], [15]. HCV replication in T lymphocytes could suppress Interferon-γ (IFN-γ)/signal transducers and activators of transcription factor 1 (STAT-1) signaling that might affect signal transducers and activators of transcription factor 3 (STAT-3) signaling[4], [13].

It has been reported that a subset of type 17 T helper (Th17) cells might be involved in various kinds of autoimmune diseases[16]–[19]. The orphan nuclear receptor RORγt (RORγt) is the key transcription factor that induces the transcription of the genes encoding Interleukin (IL)-17 in naïve CD4^+^ T helper cells[20]. Moreover, the activation of STAT-3 signaling could contribute to the induction of Th17 development[21]–[23]. Previously, Machida et al. reported that HCV replication in B lymphocytes could enhance the production of IL-6 from B lymphocyte[24]. In addition to TGF-β1, the existence of IL-6 could enhance the development of Th17 cells. IL17A-producing T lymphocytes have been recently shown to comprise a distinct lineage of pro-inflammatory T helper cells, termed Th17 cells, that are major contributors to autoimmune disease[20]. IL17A stimulates the secretion of a wide range of proinflammatory chemokines and cytokines. As its receptor is widely expressed, various kinds of immune cells as well as other cell types can respond to it[25]. Recently, we reported that the frequency of Th17 cells was remarkably high in a difficult-to-treat case of pyoderma gangrenosum-like lesion in a patient with lymphotropic HCV infection[26].

In this study, we clarified the relationship between Th17 cells and the biological significance of lymphotropic HCV.

## Material and Methods

### Study design and Patients

Two hundred-fifty patients with HCV persistent infection who were treated in Tohoku University Hospital were enrolled in this study. None of the patients had liver disease due to other causes, such as alcohol, drug, or congestive heart failure. Permission for the study was obtained from the Ethics Committee at Tohoku University Graduate School of Medicine (permission no. 2006–194) following ethical guidelines of the 1975 Declaration of Helsinki. Written informed consent was obtained from all the participants enrolled in this study. Participants were monitored for 6 months and peripheral blood samples were obtained from selected patients. We collected the peripheral blood before the treatment (treatment naïve). The concurrent diseases were diagnosed by specialized physicians belonging to the department of hematology and rheumatology. Patients were evaluated for serum levels of HCV-RNA, blood chemistry and hematology.

### Quantification of IL1β, IL6, Transforming growth factor 1 (TGF-β1) and IL17A, IL21, IL23 in the serum

The amounts of IL1β, IL6, TGF-β1, IL17A, IL21 and IL23 were quantified using IL1β, IL6, TGF-β, IL17A, IL21 and IL23 enzyme-linked immunosorbent assay (ELISA) kits (eBioscience). The serum samples from patients were collected at sampling points and stored at −20°C. The ELISA procedure was performed according to the manufacturer's protocol.

### Isolation of peripheral blood mononuclear cells (PBMCs), CD4^+^ cells, CD19^+^ cells and CD45RA^+^ naïve CD4^+^ cells

PBMCs were isolated from fresh heparinized blood by means of Ficoll-Paque (Amersham Bioscience) density gradient centrifugation. CD4^+^ T cells and CD19^+^ B cells were positively isolated by dynabeads (Dynal) to carry out the analysis of strand-specific HCV RNA detection. Naïve CD4^+^ cells were isolated by the MACS beads system (Miltenyi Biotec).

### Strand-specific intracellular HCV RNA detection

Strand-specific intracellular HCV RNA was detected using a recently established procedure that combined previously published methods [27], [28] with minor modifications [4], [13]. Positive- and negative-strand-specific HCV RNAs were detected by a nested polymerase chain reaction (PCR) method. Semi-quantification was achieved by serial fourfold dilutions (in 10 µg/ml of *Escherichia coli* tRNA) of an initial amount of 200 ng of total RNA. The relative titer was expressed as the highest dilution giving a visible band of the appropriate size on a 2% agarose gel stained by ethidium bromide. For the internal control, semi-quantification of β-actin mRNA was performed using the same RNA extracts. To rule out false, random, and self-priming, extracted HCV RNA was run in every RT–PCR test without the addition of an upstream HCV primer.

### The deep-sequencing analysis of Ly-HCV

Serum samples and PBMCs were collected from a patient with para-aortic lymph node enlargement with chronic HCV infection. Serum samples were stored at −20°C until testing. Total RNA was extracted from 800 µl of serum and 1.0×10^7^ of PBMC using Trizol LS (Invitrogen). Each library was prepared using TruSeq RNA sample preparation kits v2 (Illumina). Libraries were clonally amplified on the flow cell and sequenced on an Illumina HiSeq 2000 (HiSeq Control Software 1.5, Illumina) with a 101-mer paired end sequence. Image analysis and base calling were performed using Real Time Analysis (RTA) 1.13. In the first mapping analysis, sequence reads not of human origin were aligned with 27675 reference virus sequences registered at the Hepatitis virus database server (HVDB) (http://s2as02.genes.nig.ac.jp/index.html) and the National Center for Biotechnology Information (NCBI) (http://www.ncbi.nlm.nih.gov/) using bwa (0.5.9-r26) and allowing mismatches of within 10 nucleotide bases. Based on the highest homology to the reference virus genome in the first mapping analysis, the tentative consensus HCV full genome sequence was created. The second mapping analysis was conducted using the tentative consensus HCV full genome sequence and bwa, allowing mismatches of within 5 nucleotide bases. The result of the analysis was displayed using Integrative Genomics Viewer (IGV; 2,0,17). Sequence analysis was performed using Genetyx-Mac ver.12. A phylogenetic tree was constructed by the unweighted pair group method with the arithmetic mean. The reliability of the phylogenetic results was assessed using 100 bootstrap replicate.

### Inoculation of lymphotropic HCV strains in various kinds of lymphoid cell lines and human primary lymphocyte with stimulation

We used two different lymphotropic HCV strains. One was the SB-HCV strain that was previously reported by Sung et al[29]. The other one was Ly-HCV that was identified in this study by our group. The almost full-length sequence (95.9% coverage) of Ly-HCV was determined using a deep-sequence Hi-Seq 2000 system (illumina) (Fig S1A and B). These two-lymphotropic HCV strains were used for the experiments of HCV infection into lymphoid cells. Previously, we reported Raji, Molt-4 and primary human lymphoid cells were susceptible to the SB-HCV strain. In addition to these cells, we used miR122-transduced RIG-1/MDA-knockdown Raji cells provided by Machida K et al, since this cell line was most susceptible to SB-HCV replication (ongoing project, data not shown). These lymphotropic HCV strains were inoculated at day 0. SB cell culture supernatant and diluted serum from the patient with Ly-HCV, which contained 2×10^5^ copies/ml of HCV-RNA, were used for the infection of several kinds of human primary lymphoid cells (1×10^5^ cells). A control infection with UV-irradiated HCV was included in every experiment. The supernatant of Huh-7 cells transfected with JFH-1 strains at 10 days post-transfection was used for several control experiments. The HCV-1T strain obtained from a CH-C patient without extrahepatic diseases and lymphoproliferative diseases was also used for several control experiments.

### The analysis of IL17-secreting CD4^+^ T cells

Naïve CD4^+^ cells were negatively isolated by using a naïve CD4^+^ T cells isolation kit II (Miltenyi Biotec). Isolated naïve CD4^+^ cells were exposed to SB-HCV, Ly-HCV, UV-irradiated-SB-HCV, UV-irradiated-Ly-HCV or Mock. Then, CD3^+^CD28^+^ coated beads and various kinds of cytokines were added to the culture medium to analyze the Th17 commitment and development (Table S1). The cytokine conditions for Th17 commitment and development included IL-1β (10 ng/ml), and IL23 (1 ng/ml), which are important for the Th17 development in human, because the differentiation of Th17 cells is very difficult without these cytokines when using human PBMCs[30]. The cells were harvested at 7 days post-inoculation and IL17A-secreting cells were analyzed by MACS cytokine secretion assay (Miltenyi Biotec).

### Transwell co-culture system

The trans-membrane with 0.4 um pore size was used for the analysis of soluble factor-inducing Th17 cells, especially IL6 and TGF-β1. The upper chamber included PBMCs (2×10^6^ cells/ml) of CH-C patients (Ly-HCV or HCV-1T). The lower chamber included naïve CD4^+^ cells (2×10^5^ cells/ml) of a healthy individual and CD3CD28 coated beads with or without IL6 (40 ng/ml)(abcam) and TGF-β1 (40 ng/ml)(abcam) neutralizing antibodies. After five days incubation, the total RNA was isolated from cells of the lower chamber. The expression levels of RORγt were analyzed by real time PCR.

### Construction of Lenti-virus expressing HCV-Core antigen

HCV core cDNA cloned in pcDNA3 was kindly provided by Dr. K. Takeuchi [31]. The full length HCV core cDNA was cloned into lentiviral vector, pCSII-EF plasmid, to create the pCSII-EF-HCV core[32]. The pCSII-EF-HCV core or control pCSII-EF-IRES-GFP plasmid was transfected into HEK293T cells together with two packaging plasmids, pCAG-HIVgp and pCMV-VSV-G/RSV-Rev (provided by the RIKEN Bio-resource Center), using the calcium phosphate method. The supernatants containing the recombinant lenti-virus were used for the infection of human primary lymphocyte.

### Transfection of HCV individual protein expression plasmids

Various expression plasmids were constructed by inserting HCV-core, E1, E2, NS3, NS4B, NS5A and NS5B cDNA of genotype 1a behind the cytomegalovirus immediate-early promoter in pCDNA3.1 (Invitrogen). Primary CD4^+^ cells were transfected using 4D-Nucleofector II (Amaxa, Gaithersburg, Washington DC, USA) with a human T cell nucleofector kit (Amaxa), and various plasmids were purified using the EndFree plasmid kit (QIAGEN, Valencia, CA, USA). Viable transfected cells were isolated by Ficoll-Paque centrifugation (Amersham Bioscience) at 24 hour post-transfection. The transfection and expression efficiencies were analyzed using intracellular staining of individual proteins of HCV and flow cytometry analysis.

### Real-time PCR analysis

Cells were collected before the inoculation of lenti-virus and 10 days after the inoculation of lenti-virus. Total RNA was isolated using a column isolation kit (QIAGEN). After the isolation of RNA, one-step real-time PCR using a TaqMan Chemistry System was carried out. The ready-made set of primers and probe for the amplification of IL-6 (Hs00985639_m1), TGF-β1 (Hs00998133_m1), T-bet (Hs00203436_m1), GATA-3 (Hs00231122_m1), RORC (Hs01076112_m1) and glyceraldehyde-3-phosphate dehydrogenase (GAPDH) (Hs03929097_g1) were purchased from Applied Biosystems. The relative amount of target mRNA was obtained using a comparative threshold cycle (CT) method. The expression level of mRNAs of the non-stimulation sample of mock infected CD4^+^ cells was represented as 1.0 and the relative amounts of target mRNA were calculated according to the manufacturer's protocol.

### The analysis of STAT-1 and STAT-3 signaling

STAT-1 and STAT-3 signaling was analyzed by phospho-STAT-1 (Tyr701) and phosphor-STAT-3 (Tyr705) sandwich ELISA kit (Cell Signaling Technology). Briefly, naïve CD4^+^ cells transfected with or without HCV-core expressing plasmid were incubated with IL6 and TGF-β1. The cells were harvested at various time points. Then, the cell lysates were used for the quantification of phosphor-STAT-1 and phosphor-STAT-3.

### Statistical analysis

**Figure 1:**
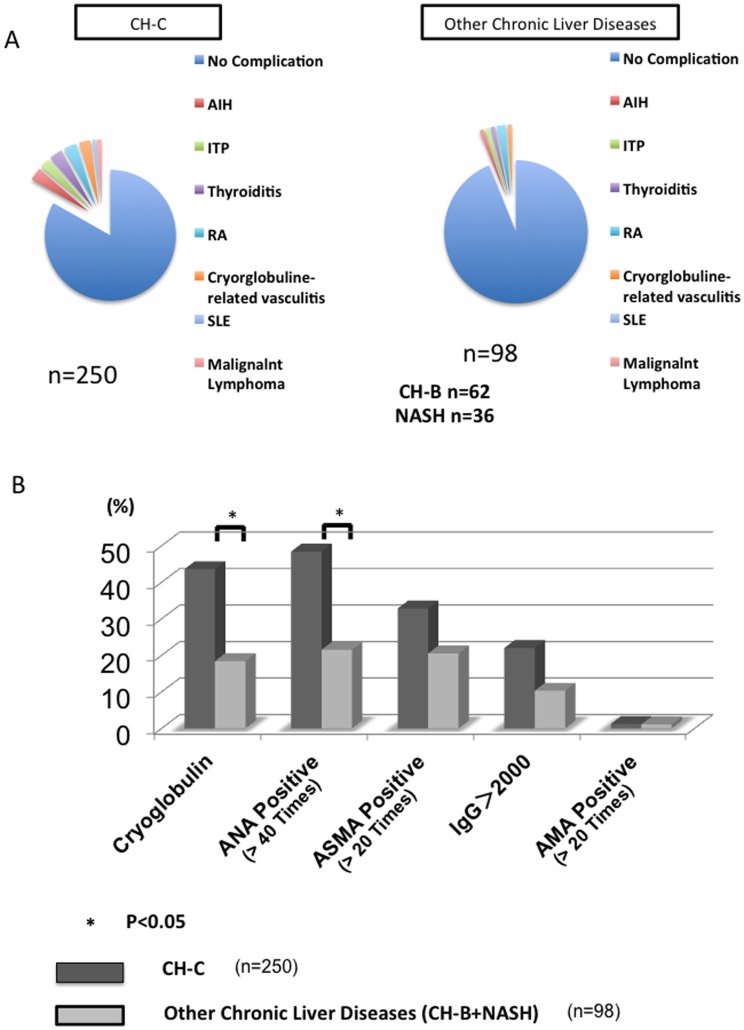
The relation between CH-C and the phenotype of autoimmune- diseases. The prevalence of these diseases in CH-C (n = 250) was significantly higher than in other chronic liver diseases (n = 98) (p = 0.0011) (A). The prevalence of these diseases and the positive rate of cryoglobulin, ANA (>40 times), ASMA (>20 times) and AMA (>20 times), and the amount of IgG (>2000mg/dl) are shown (B).

**Figure 2:**
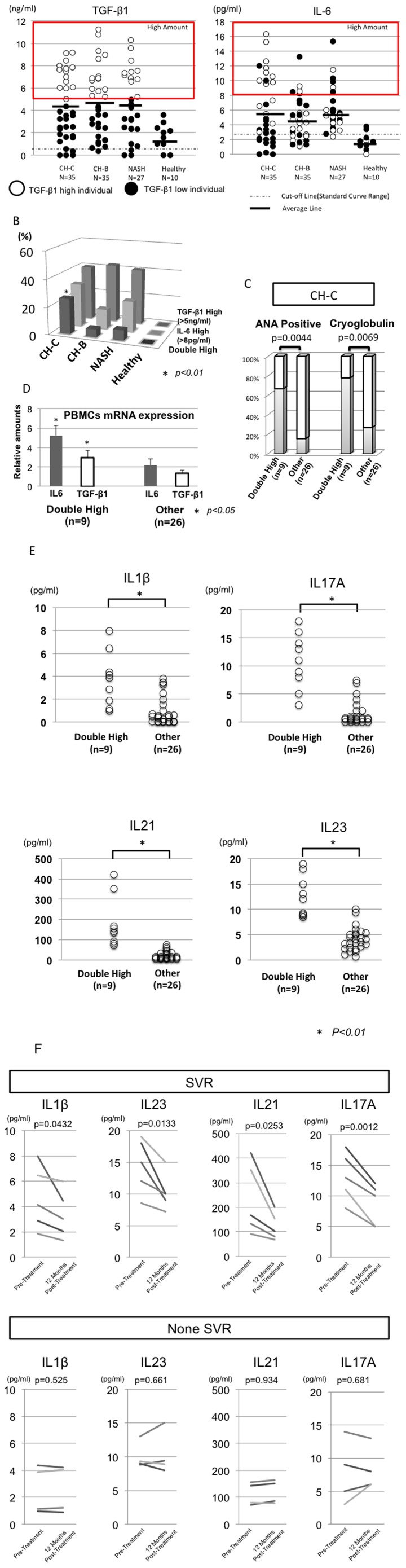
The cytokine conditions affecting the positivity of ANA and Cryoglobulin, and Th17 development. A comparison of the amounts of IL6 and TGF-β among the CH-C, CH-B, NASH and healthy subjects is shown (A). The bar indicates the mean cytokine amounts. The frequency of TGF-β1 high, IL6 high, and TGF-β1 and IL6 double high patients among the 4 groups (CH-C, CH-B, NASH, and healthy subjects) is shown (B). The positive rate of ANA and Cryoglobulin in the double high CH-C patients (n = 9) and the other CH-C patients (n = 26) is shown(C). The IL6 and TGF-β1 mRNA expression of PBMCs in the double-high patients (n = 9) and other patients (n = 26) is shown in the bar graphs (D). The amounts of IL1β, IL17A, IL21 and IL23 in the serum were compared between double high CH-C patients (n = 9) and the other CH-C patients (n = 26) (E). The comparisons of serum cytokines before and after the Peg-interferon/Ribavirin treatment are shown (F). Serum samples were collected at just before the treatment and twelve month after the end of treatment. SVR indicates sustained virological treatment (n = 5).

**Figure 3:**
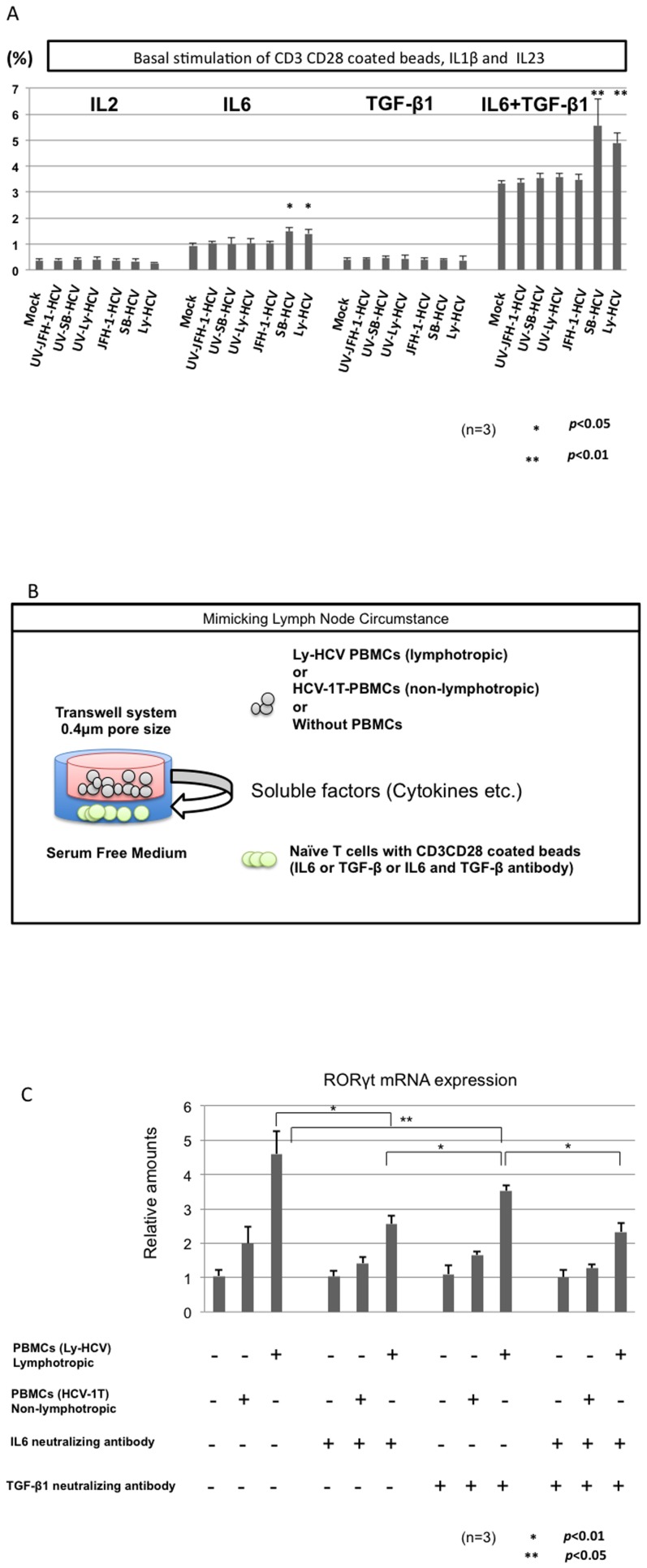
The effect of lymphotropic HCV on the Th17 development in various kinds of cytokines condition. Isolated naïve CD4^+^ cells were exposed to SB-HCV, Ly-HCV, UV-irradiated-SB-HCV, Ly-HCV or Mock. Then, CD3^+^CD28^+^ coated beads and various kinds of cytokines were added to the culture medium to analyze the Th17 commitment and development (Suppl. Table 1). The cells were harvested at 7 days post-inoculation and IL17A-secreting cells were analyzed by MACS cytokine secretion assay. The frequencies of CD4+ IL17A+ cells among the CD4+ cells were shown in the bar graph (A). The bar graph indicates the frequencies of Th17 cells with or without lymphotropic HCV in the various kinds of cytokine conditions (A). The schema of the transwell system is shown (B). The expression of RORγt mRNA in naïve T lymphocytes with or without various kinds of stimulations is shown (C). The obtained data were analyzed by Mann-Whitney U test. Three independent experiments were carried out (A)(C).

**Figure 4:**
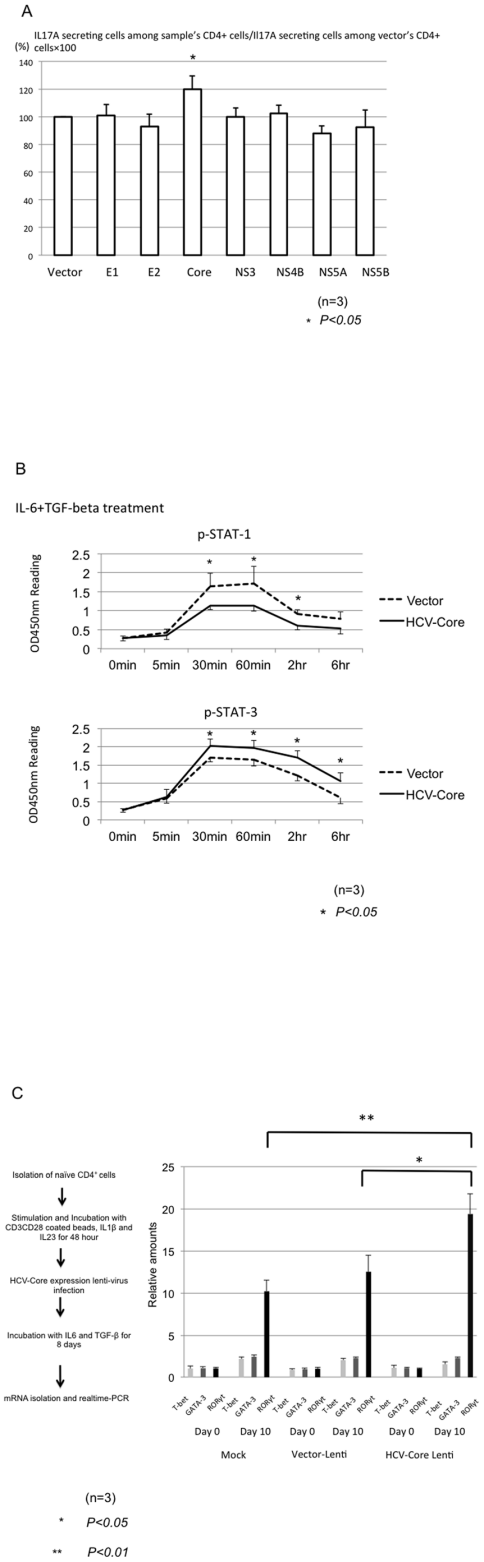
The identification of proteins responsible for enhancing the Th17 development (A). The transfection of various kinds of plasmids expressing HCV-individual proteins (E1, E2, Core, NS3, NS4B, NS5A, NS5B and vector) was carried out by nucleofector. The cells were analyzed at 72 hours post-transfection. The bar graph indicates the IL17A-secreting cells among the sample's CD4^+^ cells/IL17A secreting cells and the vector's CD4^+^ cells×100. The obtained data were analyzed by Mann-Whitney U test. Three independent experiments were carried out. **The analysis of STAT-1 and STAT-3 signaling (B).** We used a pathscan to quantify sequentially the phosphor-STAT-1 and STAT-3. The dotted lines indicate data of the vector control. Three independent experiments were carried out. **Long-term culture affected the commitment of naïve T lymphocytes with HCV-core expressing Lenti-virus (C).** The gene expressions of T-bet, GATA-3 and ROR-γt were analyzed by real-time PCR. The relative amounts of mRNA were calculated by ΔΔCT methods. The target gene expressions were analyzed at pre-inoculation of Lent-virus and 10 days after the inoculation of lenti-virus. Three independent experiments were carried out.

The data in Figure 1A, 1B, 2B and 2C were analyzed by χ^2^ test. The data in Figure 2D and 2E were analyzed by independent Students t test. Figure 3A, 3C, 4A, 4B and 4C were analyzed by Mann-Whitney U test. All statistical analyses were carried out using JMP Pro version 9.

### Accession Numbers

Accession number EntryID

AB779562 51027b2b6a8011fb860007e4.LyHCVserumSR

Accession number EntryID

AB779679 51029c6f6a8011fb8600093e.LyHCVpbmcSR

## Results

### Prevalence of autoimmune-related diseases in the CH-C patients

The prevalence of autoimmune-related disease in the CH-C patients was significantly higher than in the subjects with other chronic liver diseases in Tohoku University Hospital (p = 0.0011) (Fig.1A). In addition to the prevalence of autoimmune-related diseases, we analyzed the immunological laboratory tests including cryoglobulin, anti-nuclear antibody (ANA), anti-smooth muscle antibodies (ASMA), Immunoglobulin G (IgG), anti-mitochondrial antibody (AMA). The frequency of ANA positive or cryoglobulin positive patients in CH-C patients was significantly higher than in those with other chronic liver diseases (p<0.05) (Fig.1B).

### The amount of IL6 and TGF-β1 in the peripheral blood of CH-C patients

The average amounts of IL6 and TGF-β1 were comparable among healthy subjects, CH-C, CH-B and NASH (IL6: 1.77, 5.83, 4.84 and 5.99 pg/ml), (TGF-β: 1.45, 4.18, 4.68 and 4.5 mg/ml), (average amount) (Fig. 2A). However, the frequency of patients with high amounts of IL6 (over 8 pg/ml) and TGF-β1 (over 5 ng/ml) (double-high) was significantly higher than in those with other chronic liver diseases (*p<0.05*)(Fig. 2B). The cut-off levels of high amount of IL6 (over 8 pg/ml) and TGF-β1 (over 5 ng/ml) were determined by the appearance of two clusters(high and low) in the CH-C samples. Interestingly, Most of the TGF-β1 high CH-C patients had high amounts of IL6 (Fig. 2B). Moreover, the amount of IL6 were significantly correlated with the amount of TGF-β1(data not shown). The serum amounts of IL6 and TGFβ1 were analyzed at 6 months after the sampling points. The serum amount of IL6 and TGF-β1 in the high amount of IL6 and TGF-β1 both (double-high) patients remained doubly high (data not shown). It has been reported that the combination of IL6 and TGF-β1 cytokines could induce Th17 cells[20]. Therefore, we compared the frequency of ANA-positive or cryoglobulin-positive patients between double-high patients and the other patients with HCV persistent infection. The frequency of ANA-positive or cryoglobulin-positive patients among the double-high patients was significantly higher than among the other CH-C patients (*p<0.01*)(Fig. 2C). The expression of IL-6 and TGF-β1-mRNA in PBMCs of double-high patients was significantly higher than in other CH-C patients (*p<0.05*)(Fig. 2D). Moreover, the serum amounts of IL1-β, IL17A, IL21 and IL23 in the double-high patients were significantly higher than in the other CH-C patients (*p<0.01*) (Fig. 2E). Moreover, these cytokines were significantly correlated with the amount of IL6 and TGF-β1 (data not shown). Then, we quantified the serum cytokines at the twelve months after the Peg-interferon/Ribavirin-treatment among double high patients. The serum amounts of IL-1β, IL17A, IL21 and IL23 were significantly decreased after the achievement of the sustained virological response (SVR) (Fig. 2F).

### The relation between lymphotropic HCV and patients with high amounts of IL6 and TGF-β1 (Double-High)

Previously, Machida et al. described that HCV replication in B lymphocytes could induce their secretion of IL6. Therefore, we analyzed the relationship between lymphotropic HCV and patients with double-high by detecting strand-specific HCV-RNA in the CD4^+^ T cells and CD19^+^ B cells. The frequency of positive and negative-strand-specific-HCV-RNA in double-high CH-C patients was significantly higher than in the other CH-C patients (Table 1). These data indicated that the lymphotropism of HCV could be related to the IL6 and TGF-β1 double-high environment.

**Table 1:** The frequency of Strand specific-HCV-RNA positive CD4+ T cells and CD19+ B cells.

		Negative-st-positive		Positive-st-positive	
		%(n: positive/total)		%(n: postive/total)	
	**Double High (n = 9)**	**33.3 (3/9)**		**44.4 (4/9)**	
**CD4+ T cell**			**p = 0.0166**		**p = 0.033**
	**Other (n = 26)**	**3.8 (1/26)**		**11.5 (3/26)**	
	**Double High (n = 9)**	**44.4 (4/9)**		**66.6 (6/9)**	
**CD19+ B cell**			**p = 0.0027**		**p = 0.0003**
	**Other (n = 26)**	**3.8 (1/26)**		**7.6 (2/26)**	

St-specific HCV-RNA were detected by nested PCR with rTth polymerase.

Double high indicates that the amount of IL6 and TGF-β are high.

### Detection of a new lymphotropic HCV from a patient with lympho-proliferative disease

Previously, we used a lymphotropic SB-HCV that was reported by Sung et al[29]. In this study, we found a patient who had higher amounts of HCV RNA in the lymphocytes in comparison to other CH-C patients. This lymphotropic HCV (named Ly-HCV) is genotype 1b. The full-length sequence of this strain was analyzed by deep sequencing of both serum and PBMC samples. Phylogenetic tree analysis was then carried out (Fig. S1A). To characterize the metagenomics of HCV infection in human serum, LyHCVserumSR (registered in DDBJ; the accession number, AB779562) and PBMC, LyHCVpbmcSR (registered in DDBJ; the accession number, AB779679), we analyzed the samples by paired end deep sequencing. The coverage was 100.0% and the average depth was 2092.1× (Fig. S1B).

The LyHCVserumSR and LyHCVpbmcSR isolates were 99.5% identical to each other within the overlapping region. In 42 nucleotide bases, the major nucleotide bases showed differences. However, only the proportions of nucleotide sequences were different (Table 2). The sequences of HCV-RNA obtained from serum and PBMCs were almost the same (Table 2). Therefore, we used the diluted Ly-HCV-serum for the *in vitro* infection study.

**Table 2:** The frequency of different nucleotide bases between LyHCVserumSR and LyHCVpbmcSR.

Nucleo tide Position	LyHCVserumSR										LyHCVpbmcSR									
	Max base	No. of nucleotide	A (%)		C (%)		G (%)		T (%)		Max base	No. of nucleotide	A (%)		C (%)		G (%)		T (%)	
538	G	1835	482	(26.27)	3	(0.16)	1349	(73.51)	1	(0.05)	A	1	1	(100.0)	0	(0.00)	0	(0.00)	0	(0.00)
659	G	1821	484	(26.58)	1	(0.05)	1334	(73.26)	2	(0.11)	A	1	1	(100.0)	0	(0.00)	0	(0.00)	0	(0.00)
1,026	A	2041	1098	(53.80)	2	(0.10)	939	(46.01)	2	(0.10)	G	3	1	(33.33)	0	(0.00)	2	(66.67)	0	(0.00)
1,034	C	2120	4	(0.19)	1263	(59.58)	0	(0.00)	853	(40.24)	T	3	0	(0.00)	1	(33.33)	0	(0.00)	2	(66.67)
1,280	C	2050	2	(0.10)	1785	(87.07)	2	(0.10)	261	(12.73)	T	7	0	(0.00)	2	(28.57)	0	(0.00)	5	(71.43)
2,050	C	1595	11	(0.69)	930	(58.31)	652	(40.88)	2	(0.13)	G	4	0	(0.00)	1	(25.00)	3	(75.00)	0	(0.00)
2,105	T	1852	1	(0.05)	587	(31.70)	0	(0.00)	1264	(68.25)	C	6	0	(0.00)	4	(66.67)	0	(0.00)	2	(33.33)
2,114	A	1814	1261	(69.51)	525	(28.94)	22	(1.21)	6	(0.33)	C	7	3	(42.86)	4	(57.14)	0	(0.00)	0	(0.00)
2,136	T	1813	0	(0.00)	527	(29.07)	1	(0.06)	1285	(70.88)	C	7	0	(0.00)	4	(57.14)	0	(0.00)	3	(42.86)
2,159	T	1904	0	(0.00)	505	(26.52)	1	(0.05)	1398	(73.42)	C	7	0	(0.00)	4	(57.14)	0	(0.00)	3	(42.86)
2,234	C	2023	5	(0.25)	1128	(55.76)	3	(0.15)	887	(43.85)	T	9	0	(0.00)	3	(33.33)	0	(0.00)	6	(66.67)
2,249	C	2030	5	(0.25)	1471	(72.46)	0	(0.00)	554	(27.29)	T	8	0	(0.00)	3	(37.50)	0	(0.00)	5	(62.50)
2,717	G	1841	471	(25.58)	1	(0.05)	1365	(74.14)	4	(0.22)	A	3	2	(66.67)	0	(0.00)	1	(33.33)	0	(0.00)
3,878	T	2449	3	(0.12)	37	(1.51)	1	(0.04)	2408	(98.33)	C	3	0	(0.00)	2	(66.67)	0	(0.00)	1	(33.33)
4,043	C	1894	14	(0.74)	1836	(96.94)	0	(0.00)	44	(2.32)	T	3	0	(0.00)	1	(33.33)	0	(0.00)	2	(66.67)
4,473	C	2105	811	(38.53)	1292	(61.38)	1	(0.05)	1	(0.05)	A	5	4	(80.00)	1	(20.00)	0	(0.00)	0	(0.00)
4,661	C	2263	2	(0.09)	1301	(57.49)	1	(0.04)	959	(42.38)	T	6	0	(0.00)	0	(0.00)	0	(0.00)	6	(100.0)
5,087	G	2050	477	(23.27)	1	(0.05)	1572	(76.68)	0	(0.00)	A	5	3	(60.00)	0	(0.00)	2	(40.00)	0	(0.00)
5,114	G	1791	32	(1.79)	452	(25.24)	1306	(72.92)	1	(0.06)	C	3	0	(0.00)	2	(66.67)	1	(33.33)	0	(0.00)
5,117	A	1674	1262	(75.39)	1	(0.06)	411	(24.55)	0	(0.00)	G	3	1	(33.33)	0	(0.00)	2	(66.67)	0	(0.00)
5,156	G	1871	481	(25.71)	0	(0.00)	1389	(74.24)	0	(0.00)	A	3	2	(66.67)	0	(0.00)	1	(33.33)	0	(0.00)
5,462	C	2026	2	(0.10)	1452	(71.67)	3	(0.15)	569	(28.08)	T	11	0	(0.00)	3	(27.27)	0	(0.00)	8	(72.73)
5,535	T	2059	1	(0.05)	490	(23.80)	0	(0.00)	1568	(76.15)	C	9	0	(0.00)	5	(55.56)	0	(0.00)	4	(44.44)
5,799	G	2113	477	(22.57)	0	(0.00)	1634	(77.33)	1	(0.05)	A	3	2	(66.67)	0	(0.00)	1	(33.33)	0	(0.00)
5,804	C	2131	3	(0.14)	1613	(75.69)	3	(0.14)	511	(23.98)	T	3	0	(0.00)	1	(33.33)	0	(0.00)	2	(66.67)
5,807	T	2102	2	(0.10)	490	(23.31)	5	(0.24)	1604	(76.31)	C	3	0	(0.00)	2	(66.67)	0	(0.00)	1	(33.33)
5,831	T	2168	7	(0.32)	483	(22.28)	5	(0.23)	1672	(77.12)	C	3	0	(0.00)	2	(66.67)	0	(0.00)	1	(33.33)
5,834	T	2153	2	(0.09)	504	(23.41)	0	(0.00)	1647	(76.50)	C	3	0	(0.00)	3	(100.0)	0	(0.00)	0	(0.00)
5,837	C	2144	3	(0.14)	1674	(78.08)	2	(0.09)	465	(21.69)	T	3	0	(0.00)	1	(33.33)	0	(0.00)	2	(66.67)
5,882	T	2160	0	(0.00)	480	(22.22)	0	(0.00)	1680	(77.78)	C	3	0	(0.00)	2	(66.67)	0	(0.00)	1	(33.33)
5,883	C	2114	0	(0.00)	1502	(71.05)	1	(0.05)	611	(28.90)	T	3	0	(0.00)	1	(33.33)	0	(0.00)	2	(66.67)
5,969	T	2238	2	(0.09)	80	(3.57)	1	(0.04)	2155	(96.29)	A	1	1	(100.00)	0	(0.00)	0	(0.00)	0	(0.00)
5,978	C	2451	1	(0.04)	2013	(82.13)	1	(0.04)	436	(17.79)	T	1	0	(0.00)	0	(0.00)	0	(0.00)	1	(100.0)
7,172	C	2409	2	(0.08)	1696	(70.40)	1	(0.04)	707	(29.35)	T	3	0	(0.00)	1	(33.33)	0	(0.00)	2	(66.67)
7,274	A	2654	1564	(58.93)	5	(0.19)	1074	(40.47)	11	(0.41)	G	7	3	(42.86)	0	(0.00)	4	(57.14)	0	(0.00)
7,349	G	2510	1019	(40.60)	5	(0.20)	1484	(59.12)	2	(0.08)	A	6	4	(66.67)	0	(0.00)	2	(33.33)	0	(0.00)
7,932	G	2810	1354	(48.19)	0	(0.00)	1453	(51.71)	3	(0.11)	A	9	7	(77.78)	0	(0.00)	2	(22.22)	0	(0.00)
8,093	G	2647	684	(25.84)	3	(0.11)	1956	(73.89)	4	(0.15)	A	7	4	(57.14)	0	(0.00)	3	(42.86)	0	(0.00)
8,168	G	1993	39	(1.96)	1	(0.05)	1952	(97.94)	1	(0.05)	A	2	1	(50.00)	0	(0.00)	1	(50.00)	0	(0.00)
8,237	C	2077	1	(0.05)	2033	(97.88)	0	(0.00)	42	(2.02)	T	3	0	(0.00)	1	(33.33)	0	(0.00)	2	(66.67)
8,672	G	2340	17	(0.73)	576	(24.62)	1734	(74.10)	12	(0.51)	C	3	0	(0.00)	2	(66.67)	1	(33.33)	0	(0.00)
8,693	T	2899	5	(0.17)	690	(23.80)	0	(0.00)	2204	(76.03)	C	3	0	(0.00)	2	(66.67)	0	(0.00)	1	(33.33)

### Analysis of Infectivity of Ly-HCV and SB-HCV under the various kinds of cytokines

We examined the infectivity of Ly-HCV and SB-HCV into several lymphoid-cell lines (Raji, miR122-transduced RIG-1/MDA-knockdown Raji, and Molt-4) and primary naïve CD4^+^ T cells. Semi-quantitative strand-specific nested PCR was carried out as in our previous reports (Table 3). The infectivity of HCV in the IL-6 and TGF-β cytokine combination conditions with low dose IL1β, IL23 and CD3CD28 coated beads was no better than that in IL2, IL6, or TGF-β cytokine only conditions with low dose IL1β, IL23 and CD3CD28 coated beads.

**Table 3:** Detection of St-Specific HCV-RNA in various kinds of lymphoid cell.

Immune cells	Raji				mir122Raji				Molt-4				naïve T (IL-2)				naïve T (IL6)				naïve T (TGF-β)				naïveT (IL6 and TGF-β)			
HCV-Strain	JFH	HCV	SB	Ly	JFH	HCV	SB	Ly	JFH	HCV	SB	Ly	JFH	HCV	SB	Ly	JFH	HCV	SB	Ly	JFH	HCV	SB	Ly	JFH	HCV	SB	Ly
																												
Positive Strand																												
2 days	1	1	1	1	1	1	1	1	1	1	1	1	1	1	1	1	1	1	1	1	1	1	1	1	1	1	1	1
7 days	0	1	16	4	0	1	64	16	0	1	16	4	0	0	16	4	0	0	4	4	0	0	16	4	0	0	16	4
7 days-UV-irraidated	0	0	0	0	0	0	0	0	0	0	0	0	0	0	0	0	0	0	0	0	0	0	0	0	0	0	0	0
																												
Negative Strand																												
2 days	0	0	1	0	0	0	1	1	0	0	0	0	0	0	1	0	0	0	0	0	0	0	0	0	0	0	0	0
7days	0	0	4	1	0	0	16	4	0	0	4	1	0	0	4	1	0	0	1	1	0	0	4	1	0	0	4	1
7days-UV-irradiated	0	0	0	0	0	0	0	0	0	0	0	0	0	0	0	0	0	0	0	0	0	0	0	0	0	0	0	0

The titers of HCV-RNA were expressed as the highest dilution giving a visible band of the correct size.

Naïve T cells were incubated with IL-1β (10 ng/ml), IL23 (1 ng/ml), and CD3CD28 coated beads.

JFH-1 and HCV-1T are not lymphotropic HCV strains.

SB-HCV and Ly-HCV are lymphotropic HCV strains.

mir122Raji indicate miR122-transduced RIG-1/MDA-knockdown Raji.

### The effect of lymphotropic HCV on the Th17 development

The addition of both IL6 and TGF-β1 could significantly induce IL17-secreting T cells (Th17) in comparison to IL6 or TGF-β1 alone (Fig 3A). Both lymphotropic HCV strains (SB-HCV and Ly-HCV) could significantly up-regulate the Th17 development in comparison to Mock and these strains that had been UV-irradiated. Then, we used a co-culture system to analyze the blocking of IL6 and TGF-β1 effects since the expressions of IL6 and TGF-β1 mRNA in PBMCs of double high patients were significantly higher than those in other CH-C patients (Fig. 2D and Fig. 3B). The IL-6 was produced from B lymphocytes. Moreover, the major TGF-β1 producing cells were monocytes in double high patients (Data not shown). The soluble factors produced from PBMCs of Ly-HCV-patient could significantly induce Th17 master gene RORγt in comparison to mock and PBMCs of HCV-1T patient (Fig. 3C). The addition of IL6 and TGF-β1 neutralizing antibody significantly reduced the expression of RORγt, especially IL6 neutralizing antibody (Fig. 3C).

### The identification of HCV proteins and signal transduction responsible for the production of IL17A

We used E1, E2, Core, NS3, NS4B, NS5A and NS5B expressing plasmids to transiently express these proteins in naïve T cells. The transfection efficiencies were 45.4±4.96% (average±standard deviation). Among these proteins, only HCV-Core protein could significantly enhance the production of IL17A cells (Fig. 4A)(p<0.05). In addition to in vitro circumstance, we used NOD/scid/γc^null^ (NOG) mice that are super-immunodeficiency mice[33]. The transfusions of HCV-core expressing human primary lymphoid cells were carried out (*ongoing Kondo Y et al.*). The higher amount of IL17A and RORγt mRNA were detected in the HCV-Core expressing CD4^+^ cells in comparison to the control groups (data not shown). Then, we sequentially analyzed the STAT-1 and STAT-3 activation by IL6 and TGF-β1 stimulation in the HCV-Core expressing T cells. The results indicated that STAT-3 signaling was significantly enhanced in comparison to mock-transfected T cells (Fig. 4B)(p<0.05). These data indicated that HCV-Core protein enhanced the STAT-3 signaling following the induction of the Th17 master gene-RORγt.

### Long-term culture of primary naïve T lymphocytes with HCV-core expressing lenti-virus

We constructed the HCV-core expressing lenti-virus to analyze the long-term culture of primary naïve T lymphocytes with the expression of HCV core protein. The efficiency of lenti-virus infection was 27.7±3% (average±standard deviation). In addition to IL1β and IL23, the IL6 and TGF-β1 cytokine conditions could remarkably induce the RORγt mRNA (Fig.4C). Moreover, significantly higher amounts of RORγt mRNA were detected in the HCV-core expressing T lymphocytes in comparison with the control groups (Fig. 4C)(p<0.05).

## Discussion

Autoimmune thyroiditis, systemic lupus erythematosus, idiopathic thrombocytopenic purpura, autoimmune hepatitis and rheumatoid arthritis etc. could be classified not only as HCV-related diseases but also as Th17-related autoimmune diseases[16]-[19], [34]–[36]. In this study, we clearly demonstrated the relevance of lymphotropic HCV to autoimmune-related diseases including an important role of Th17 cells in CH-C patients. This study revealed two important mechanisms by which Th17 development is enhanced. In the first, the existence of lymphotropic HCV can result in the IL6 and TGF-β1 double high condition that can enhance Th17 development. Previously, Machida et al. described that HCV replication in B cells induced IL6 production from B cells[24]. In the second, the existence of HCV in naïve T cells can enhance Th17 development in the IL6 and TGF-β1 double high condition by enhancing STAT-3/RORγt signaling. Previously, we showed that lymphotropic HCV could suppress IFN-γ/STAT-1/T-bet signaling, which could contribute to the persistence of hepatitis C virus infection[1], [4], [13]. STAT-3 signaling could be enhanced by the suppression of STAT-1 signaling. However, the finding of our research was surprising. Therefore, we examined this phenomenon studiously and carefully. First, we found a novel genotype, 1b lymphotropic HCV strain (Ly-HCV), that could infect Raji and human primary lymphocytes. Although the infectivity of this strain was lower than that of SB-HCV[29], we could detect negative and positive strand RNA in naïve T lymphocytes with stimulation.

Therefore, we used two lymphotropic HCV strains to analyze the effect on Th17 development. Moreover, two kinds of expression experiments showed that HCV-Core could enhance the STAT-3/RORγt signaling, since the method of gene expression and the period of incubation might affect the result of T cell development. However, the results of our two kinds of experiments (plasmids and lenti-virus) consistently showed that the expression of HCV-core protein in T lymphocytes could enhance the Th17 development. Other groups previously reported that HCV-core protein could affect anergy-related genes and T cell responses by inducing spontaneous and alternating T-cell receptor-triggered Ca2+ oscillations[37], [38]. Therefore, the expression of HCV-core protein in T lymphocyte might be important for the functional changes in T lymphocytes. Although our study confirmed that the replication of HCV in lymphocytes is important, there was a bystander effect by exosome produced from HCV-infected lymphocytes. Previously, our group reported that exosome could transport miRNA and proteins in the microenviroment[39], [40]. This phenomenon could explain the significant effects of a low level of HCV infection in lymphocytes. Moreover, the naïve T lymphocyte is located upstream of Th17 development. Therefore, we should not underestimate the effect of a low-level of HCV replication in lymphocytes.

In conclusion, we report the detailed mechanism of Th17 development and HCV infection, which might be involved in the pathogenesis of autoimmune-related disease in CH-C patients (Fig S2). Recently, a novel therapy targeting STAT-3 signaling was reported[23], [41], [42]. We should consider the clinical use of such treatments for autoimmune-related diseases in CH-C patients.

## Supporting Information

Figure S1 **Phylogenetic trees constructed based on the nearly entire nucleotide sequence of HCV by using the unweighted pair group method with the arithmetic mean (Michener 1957).** The tree includes the three genotype 1a isolates, forty 1b, one 1c, three 2a, two 2b, one 2c and one 3a, 3b, 6b, 7b, 9b, 10a, whose nucleotide sequence data were retrievable from the GenBank/EMBL/DDBJ database (A). Mapping to the consensus HCV genome sequence. For Ly-HCV 0183-4, 197,414 reads were mapped (Fig S1B). The coverage was 100.0%, and the average depth was 2092.1x. For Ly-HCV 0186-1, 410 reads were aligned. The coverage was 95.9%, and the average depth was 4.3× (B).
(TIFF)

Figure S2 **The schema of Th17 induction in perihepatic lymph node of CH-C patient with lymphotropic HCV are shown.**
(TIFF)

Table S1 **Various kinds of cytokines conditions for in vitro analysis are shown in Table S1.**
(DOC)
