# Supplementary figures and images for: HCV Infection Enhances Th17 Commitment, Which Could Affect the Pathogenesis of Autoimmune Diseases
Source: PLoS One. 2014 Jun 6;9(6):e98521. doi: 10.1371/journal.pone.0098521 (PMC4048196; doi:10.1371/journal.pone.0098521)

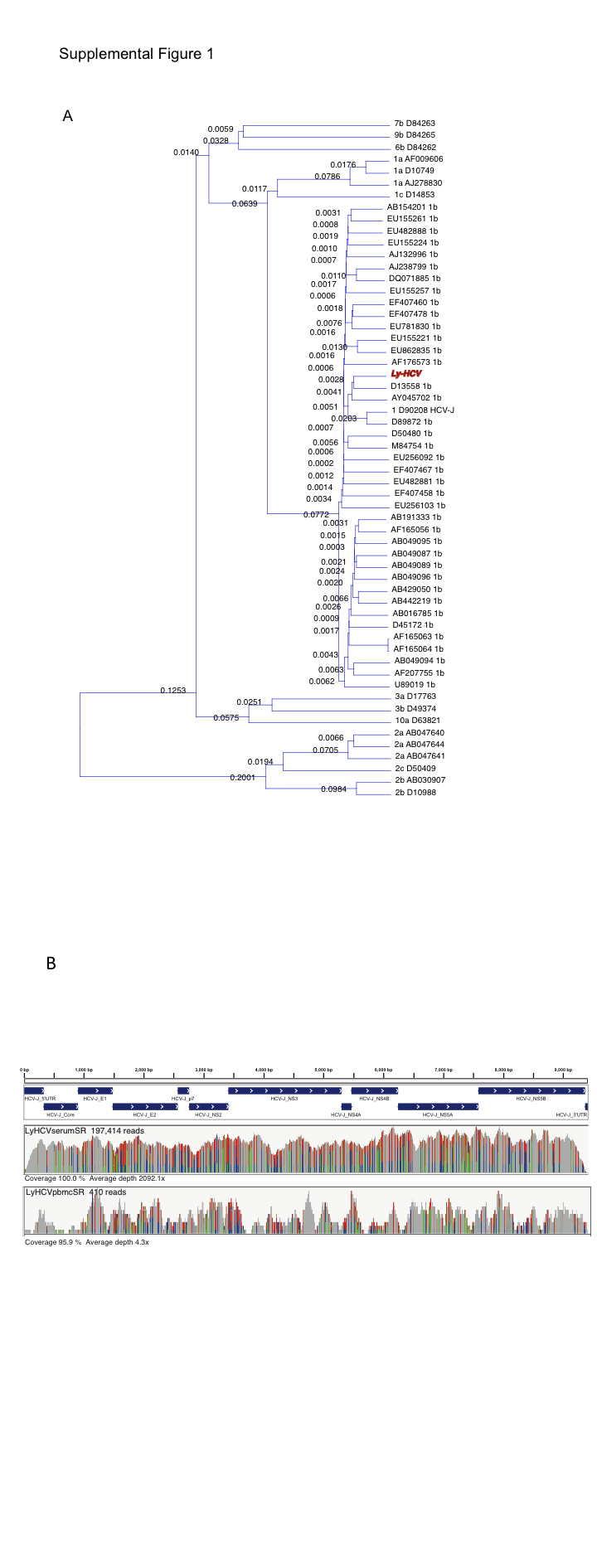

Supplement: Figure S1 — Phylogenetic trees constructed based on the nearly entire nucleotide sequence of HCV by using the unweighted pair group method with the arithmetic mean (Michener 1957). The tree includes the three genotype 1a isolates, forty 1b, one 1c, three 2a, two 2b, one 2c and one 3a, 3b, 6b, 7b, 9b, 10a, whose nucleotide sequence data were retrievable from the GenBank/EMBL/DDBJ database (A). Mapping to the consensus HCV genome sequence. For Ly-HCV 0183-4, 197,414 reads were mapped (Fig S1B). The coverage was 100.0%, and the average depth was 2092.1x. For Ly-HCV 0186-1, 410 reads were aligned. The coverage was 95.9%, and the average depth was 4.3× (B). (TIFF) [file pone.0098521.s001.tif]

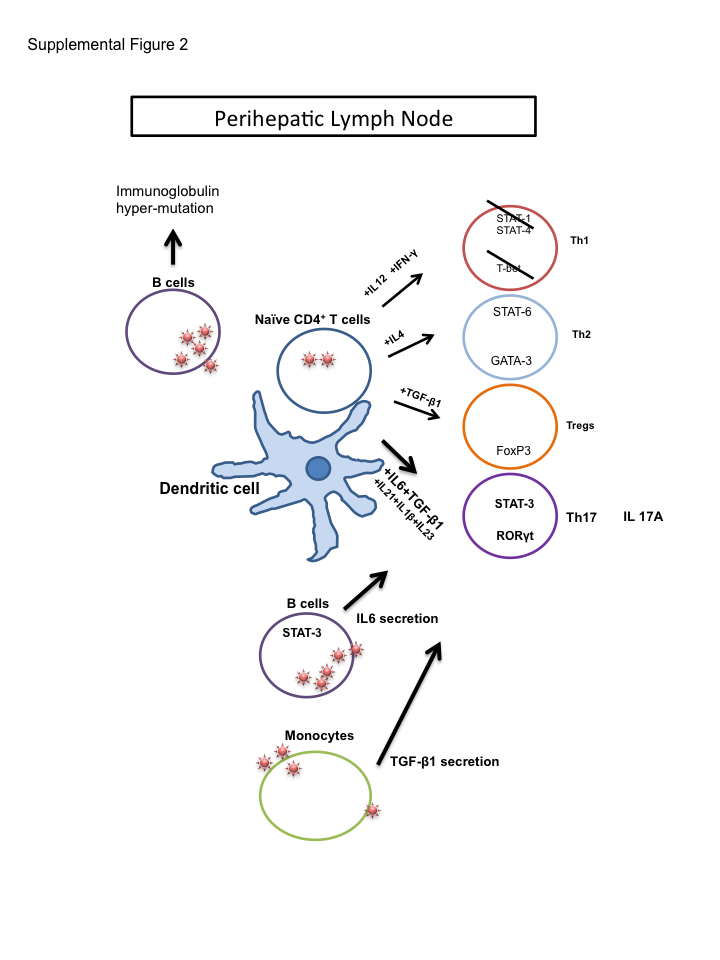

Supplement: Figure S2 — The schema of Th17 induction in perihepatic lymph node of CH-C patient with lymphotropic HCV are shown. (TIFF) [file pone.0098521.s002.tif]
