# Supplementary material for: HCV Infection Enhances Th17 Commitment, Which Could Affect the Pathogenesis of Autoimmune Diseases
Source: PLoS One. 2014 Jun 6;9(6):e98521. doi: 10.1371/journal.pone.0098521 (PMC4048196; doi:10.1371/journal.pone.0098521)
Supplement: Supplementary file 2 [file pone.0098521.s003.doc]

Supplemmental Table 1

Various kinds of cytokines conditions for *in vitro*

Day0	Day2	Day5	Day7
HCV-Inoculation(2×10^5 copies)<br>IL1-β(10ng/ml) and IL23 (1ng/ml)<br>CD3+CD28+ beads	IL2(50ng/ml)<br>IL1-β(10ng/ml) and IL23 (1ng/ml)	IL2(50ng/ml)<br>IL1-β(10ng/ml) and IL23 (1ng/ml)	Harvest<br>Analysis
HCV-Inoculation(2×10^5 copies)<br>IL1-β(10ng/ml) and IL23 (1ng/ml)<br>CD3+CD28+ beads	IL6(20ng/ml)<br>IL1-β(10ng/ml) and IL23 (1ng/ml)	IL6(20ng/ml)<br>IL1-β(10ng/ml) and IL23 (1ng/ml)	Harvest<br>Analysis
HCV-Inoculation(2×10^5 copies)<br>IL1-β(10ng/ml) and IL23 (1ng/ml)<br>CD3+CD28+ beads	TGF-β(5ng/ml)<br>IL1-β(10ng/ml) and IL23 (1ng/ml)	TGF-β(5ng/ml)<br>IL1-β(10ng/ml) and IL23 (1ng/ml)	Harvest<br>Analysis
HCV-Inoculation(2×10^5 copies)<br>IL1-β(10ng/ml) and IL23 (1ng/ml)<br>CD3+CD28+ beads	IL6(20ng/ml)+TGF-β (5ng/ml)<br>IL1-β(10ng/ml) and IL23 (1ng/ml)	IL6(20ng/ml)+TGF-β(5ng/ml)<br>IL1-β(10ng/ml) and IL23 (1ng/ml)	Harvest<br>Analysis
